# Long Working Hours and Risk of Nonalcoholic Fatty Liver Disease: Korea National Health and Nutrition Examination Survey VII

**DOI:** 10.3389/fendo.2021.647459

**Published:** 2021-05-06

**Authors:** Eyun Song, Jung A. Kim, Eun Roh, Ji Hee Yu, Nam Hoon Kim, Hye Jin Yoo, Ji A. Seo, Sin Gon Kim, Nan Hee Kim, Sei Hyun Baik, Kyung Mook Choi

**Affiliations:** Division of Endocrinology and Metabolism, Department of Internal Medicine, Korea University College of Medicine and School of Medicine, Seoul, South Korea

**Keywords:** fatty liver disease, hepatic steatosis index, occupational health, metabolic diseases, liver steatosis

## Abstract

**Background:**

The global incidence of NAFLD is rising sharply due to various risk factors. As previous studies reported adverse health impact of long working hours on metabolic diseases, such as diabetes mellitus and obesity, it is plausible that NAFLD is also associated with working excessive hours. However, data regarding this issue is limited.

**Methods:**

In this cross-sectional study based on Korea National Health and Nutrition Examination Survey VII, 5,661 working adults without previous liver disease or heavy alcohol drinking habits were included. The subjects were categorized into three groups according to working hours: 36–42, 43–52, and 53–83 hours/week. NAFLD was defined using the hepatic steatosis index (HSI), which is a validated prediction model for determining NAFLD.

**Results:**

The prevalence of NAFLD (HSI ≥36) increased with longer working hours: 23.0%, 25.6%, and 30.6% in the 36–42, 43–52, and 53–83 hours/week group, respectively (p <0.001). Subjects who worked 53–83 hours/week had higher odds for NAFLD than those who worked the standard 36–42 hours/week (OR 1.23, 95% CI 1.02–1.50, p = 0.033) after adjusting for age, sex, body mass index, smoking, alcohol, exercise, diabetes mellitus, hypertension, serum triglyceride, and total cholesterol. This association was consistent across subgroups according to working schedule (daytime *vs.* shift workers) or occupation type (office *vs.* manual workers). In particular, the relationship between long working hours and NAFLD was pronounced in workers aged <60 years and in female workers.

**Conclusions:**

Long working hours was significantly associated with NAFLD. Further prospective studies are required to validate this finding with causal relationship.

## Introduction

Working long hours is an emerging critical health issue worldwide, threatening the health of workers in various aspects. Previous studies have reported its association with cardiovascular disease (1, 2), diabetes mellitus (3), metabolic syndrome (4), and obesity (5). Although the International Labour Office had set a general standard of 48 hours/week with a maximum of 8 hours/day, a substantial portion of workers work over this limit (6), resulting in increased work-related health problems.

NAFLD is a leading cause of liver disease with a global prevalence of ~ 25% (7, 8). The disease burden of NAFLD is expected to rise sharply worldwide as the incidence of obesity, diabetes mellitus, and metabolic syndrome—conditions with a well-established association with NAFLD—is increasing (9). As working long hours heightened the risk of such conditions, it may also increase the risk of NAFLD. However, to our knowledge, no previous studies have reported the association between long working hours and NAFLD. To fill this research gap, this study aimed to assess the relationship between long working hours and NAFLD by analyzing data from a large population-level survey.

## Materials and Methods

### Study Design and Population

Data were obtained from the Korea National Health and Nutrition Examination Survey (KNHANES), a nationwide cross-sectional survey conducted by the Korea Centers for Disease Control and Prevention to provide national health, diet, and nutrition data representing the civilian, noninstitutionalized Korean population. Data from version VII, the latest survey conducted from 2016 to 2018, were used for this study. Research subjects were selected using two-stage stratified cluster sampling of the population and housing census data. The data were collected *via* household interviews and standardized physical examinations. Informed consent was obtained from each participant before performing the survey, and secondary anonymized data were used in the analyses. The Korea University Institutional Review Board approved the study protocol in accordance with the Declaration of Helsinki by the World Medical Association.

A total of 10,677 subjects aged 19 – 80 years who are currently working were initially screened (**Supplementary Figure 1**). Subjects who met the following criteria were excluded: 1) unavailable data on working hours or working schedule; 2) engaged in part-time work (<36 hours/week, according to the criteria of the Korea Labor Institute (10)) or in extremely long working hours (>83 hours/week, far exceeding the legal limits); 3) previous diagnosis of hepatitis B, hepatitis C, or liver cirrhosis; 4) heavy alcohol consumption (≥2 drinks per week with an average of ≥7 alcohol units at a time for men and ≥5 alcohol units at a time for women); and 5) any missing parameter for calculating the hepatic steatosis index (HSI). Finally, 5,661 subjects were included in the study, representing 14,121,493 Koreans.

### NAFLD

The presence of NAFLD was assessed using HSI. HSI is a well-validated prediction model for determining NAFLD and is calculated using the following formula: HSI = 8 alanine aminotransferase/aspartate aminotransferase ratio + body mass index (BMI) + 2 if diabetes + 2 if female (11). An HSI index of > 36 was considered to indicate the presence of NAFLD. Previous studies have validated this scoring system using liver biopsy, abdominal ultrasonography, proton magnetic resonance, and controlled attenuation parameter measurement in large samples, and showed a good correlation between HSI and the degree of NAFLD (12–14).

### Working Patterns

KNHANES collects information on occupation through an interview conducted by a trained medical staff and interviewers (15). The subjects disclose their average working hours per week including overtime and night shifts as an open question. They also provide their working schedule by choosing among 1) working in daytime (between 6 am and 6 pm), 2) working in the afternoon (2 pm to midnight), 3) working at night (9 pm to 8 am the next day), 4) work in rotation shifts (day to night), 5) work on alternate days, 6) work in split shifts (more than two spate timeslot a day), or 7) irregular shifts. Then the subjects answer whether they work as managers, professionals or related experts, clerks, service workers, sales workers, agricultural/forestry/fishery workers, skilled workers, device and machine operators, or elementary manual workers. Subjects also disclose whether they are self-employed, employees, or unpaid family workers.

Subjects were grouped according to their working hours by 36–42, 43–52, and 53–83 hours/week, as previously described (16). They were also categorized to “daytime workers” if they worked mainly between 6 am and 6 pm and to “shift workers” if they worked in any other schedule for subgroup analyses. Furthermore, occupations were grouped into “office work” (managers, professionals and related experts, clerks, service workers, and sales workers) and “manual work” (agricultural/forestry/fishery workers, skilled workers, device and machine operators, and elementary manual workers).

### Statistical Analysis

R version 3.4.0 software and the R libraries survey, RODBC, car, and Cairo were used for data analysis (R Foundation for Statistical Computing, Vienna, Austria; available at http://www.R-project.org). All statistics were calculated using sample weights assigned to sample participants by the KNHANES. The sample weights were constructed to attain unbiased estimates representing the entire Korean population, with consideration of the stratified multistage probability sampling design of each survey year. Continuous variables are presented as medians with interquartile ranges, and categorical variables are presented as frequencies with weighted percentages. The chi-square test was used to compare the prevalence of NAFLD according to working hours. Associations between NAFLD and working hours were analyzed using multivariable logistic regression models adjusting for confounding factors such as age, sex, smoking, alcohol, BMI, comorbidities (diabetes mellitus ****defined as previously diagnosed as diabetes mellitus or receiving glucose-lowering treatment, and hypertension defined as previously diagnosed as hypertension or receiving blood pressure-lowering medication****), serum triglyceride, and serum total cholesterol. Differences with p <0.05 were considered statistically significant.

## Results

### Baseline Characteristics of the Study Subjects

The baseline characteristics of the study subjects according to the presence of NAFLD are summarized in **Table 1**. Among 5,661 subjects, 1393 (24.6%) had NAFLD based on HSI. The median age was 46 years in both groups. Men accounted for 56.6% of the subjects without NAFLD but accounted for a significantly higher proportion (74.6%) of those with NAFLD (p <0.001). Higher BMI and higher prevalence of diabetes mellitus or hypertension were observed in subjects with NAFLD than in those without. The median working hours per week were 47 hours/week in the NAFLD group and 45 hours/week in the non-NAFLD group. Significant differences were noted in working schedule and type of employment but not in occupation type between the two groups. Subjects without NAFLD slept longer hours compared with those with NAFLD.

**Table 1:** Baseline characteristics.

	NAFLD (-)(n = 4,268)	NAFLD (+)(n = 1,393)	*p*-value
Age, years	46.0 [36.0–57.0]	46.0 [37.0–55.0]	0.331
Sex			<0.001
Male	2138 (56.6%)	932 (74.6%)	
Female	2130 (43.4%)	461 (25.4%)	
Alcohol			0.158
No	275 (5.1%)	100 (6.2%)	
Yes	3993 (94.9%)	1293 (93.8%)	
Smoking			<0.001
Never	2490 (54.1%)	651 (42.4%)	
Ex-smoker	956 (23.7%)	352 (26.7%)	
Current smoker	822 (22.3%)	390 (30.9%)	
Regular exercise*			0.748
No	763 (17.9%)	255 (18.3%)	
Yes	3505 (82.1%)	1138 (81.7%)	
BMI	22.7 [20.9–4.4]	27.6 [25.9–29.6]	<0.001
Fasting glucose (mg/dL)	93.0 [88.0–100.0]	100.0 [93.0–113.0]	<0.001
HbA1c (%)	5.4 (5.2–5.7)	5.7 (5.4–6.1)	<0.001
Total cholesterol (mg/dL)	191.0 [168.0–215.0]	199.0 [173.0–224.0]	<0.001
Total triglyceride (mg/dL)	94.5 [67.0–138.0]	147.0 [100.0–213.0]	<0.001
Systolic blood pressure	112.0 [104.0–123.0]	121.0 [112.0–130.0]	<0.001
Diastolic blood pressure	74.0 [69.0–81.0]	80.0 [75.0–87.0]	<0.001
Diabetes mellitus (yes)	146 (2.9%)	162 (9.6%)	<0.001
Hypertension (yes)	525 (10.3%)	375 (22.4%)	<0.001
Working hours per week	45.0 [40.0–53.0]	47.0 [40.0–55.0]	<0.001
36–42	1767 (40.4%)	492 (34.6%)	
43–52	1414 (34.6%)	471 (34.0%)	
53–83	1087 (24.9%)	430 (31.4%)	
Working schedule			<0.001
Daytime	3714 (87.0%)	1200 (86.1%)	
Afternoon	176 (4.1%)	81 (5.8%)	
Night	59 (1.4%)	36 (2.6%)	
Regular shifts	292 (6.8%)	71 (5.1%)	
Irregular shifts	27 (0.6%)	5 (0.4%)	
Type of employment			0.047
Self-employed	845 (19.8%)	307 (22.0%)	
Employee	3240 (75.9%)	1013 (72.7%)	
Unpaid family worker	183 (4.3%)	73 (5.2%)	
Occupation			0.062
Office worker	2922 (69.7%)	913 (66.8%)	
Manual worker	1346 (30.3%)	480 (33.2%)	
Sleep duration			0.02
<5 hours/day	145 (3.4%)	64 (4.6%)	
5–6 hours/day	1564 (36.6%)	543 (39.0%)	
≥7 hours/day	2559 (60.0%)	786 (56.4%)	

BMI, body mass index.

*Regular exercise was defined as either walking or weight training for more than two days a week on average.

Working patterns and sleep duration according to three groups based on working hours (36–42, 43–52, and 53–83 hours/week) are presented in **Supplementary Table 1**. Subjects working 36–42 hours/week had higher proportion of subjects working at daytime (89.0%) than those working 43–52 (88.0%) or 53–83 (82.1%) hours/week. In addition, subjects working long hours were mostly self-employed as 45.9% of the self-employed subjects worked 53–83 hours/week and only 25.1% of them worked standard hours (36–42 hours/week). On the other hand, in employees, most of them (42.3%) worked standard hours and only 21.4% of them worked over 53 hours/week. As expected, subjects working long hours had shorter duration of sleep than those working standard hours.

### Prevalence of NAFLD According to Working Hours

**Figure 1:**
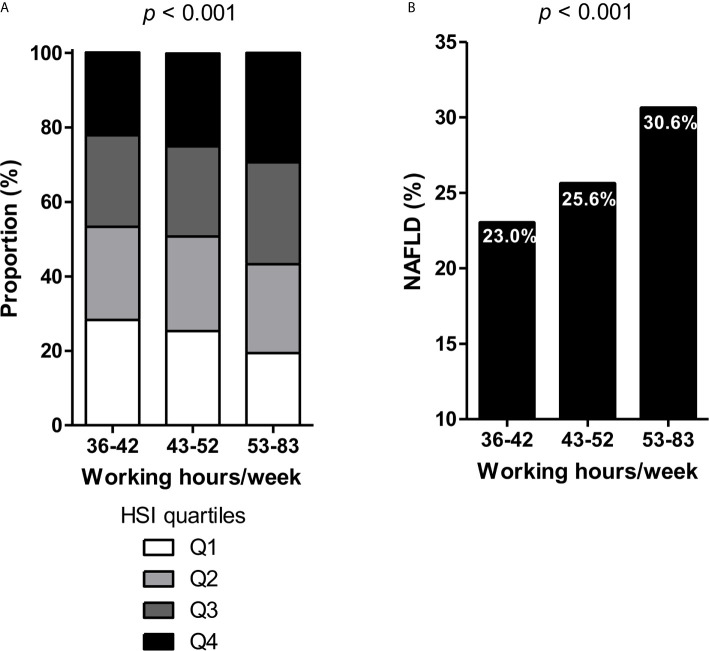
**(A)** Hepatic steatosis index (HSI) by quartiles and **(B)** prevalence of NAFLD according to working hours.

A strong association was observed between working hours and HSI by quartiles based on working hours (p <0.001, **Figure 1A**). Similar results were observed for BMI, aspartate aminotransferase, and alanine aminotransferase levels by quartiles in relation to working hours (all p <0.001, **Supplementary Figures 2A–C**). By using the cutoff level of 36, the prevalence of NAFLD (HSI ≥36) also significantly differed among the three groups: 23.0%, 25.6%, and 30.6% of subjects with NAFLD in the 36–42, 43–52, and 53–83 hours/week groups, respectively (**Figure 1B**).

### Association Between Working Hours and NAFLD

Multivariable logistic regression was performed to assess the association between working hours and NAFLD after adjusting for common confounding covariates for NAFLD (**Table 2**). The minimally adjusted model with age and sex (model 1) showed a significantly higher OR for NAFLD of 1.34 (95% CI 1.13–1.59, p <0.001) in the 53–58 hours/week group than the 36–42 hours/week group as the reference. The association was also significant after adjusting for age, sex, smoking, alcohol, exercise, BMI, history of previous diabetes mellitus or hypertension, serum triglyceride, and serum total cholesterol with an OR of 1.23 (95% CI 1.02–1.50, p = 0.033; model 2). After further adjusting for working schedule (daytime *vs.* afternoon *vs.* night *vs.* regular shifts *vs.* irregular shifts), type of employment (self-employed vs. employee vs. unpaid family worker), and sleep duration (<5 hours/day *vs.* 5–6 hours/day *vs.* ≥7 hours/day), the 53–58 hours/week group still had higher OR for NAFLD of 1.23 (95% CI 1.00–1.50, p = 0.046; model 3).

**Table 2:** Association between working hours and NAFLD.

	NAFLD assessed using HSI		
	OR	95% CI	*p*-value
Model 1			
36–42 hours/week	Ref	–	
43–52 hours/week	1.07	0.91–1.27	0.405
53–83 hours/week	1.34	1.13–1.59	< 0.001
Model 2			
36–42 hours/week	Ref	–	
43–52 hours/week	1.05	0.86–1.28	0.630
53–83 hours/week	1.23	1.02–1.50	0.033
Model 3			
36–42 hours/week	Ref	–	
43–52 hours/week	1.06	0.87–1.30	0.557
53–83 hours/week	1.23	1.00–1.50	0.046

HSI, hepatic steatosis index.

Model 1: adjusted for age and sex.

Model 2: adjusted for age, sex, smoking, alcohol, exercise, body mass index, diabetes mellitus, hypertension, serum triglyceride, and serum total cholesterol.

Model 3: adjusted for age, sex, smoking, alcohol, exercise, body mass index, diabetes mellitus, hypertension, serum triglyceride, and serum total cholesterol, working schedule (daytime vs. afternoon vs. night vs. regular shifts vs. irregular shifts), type of employment (self-employed vs. employee), and sleep duration (< 5 hours/day vs. 5–6 hours/day vs. ≥7 hours/day).

**Figure 2:**
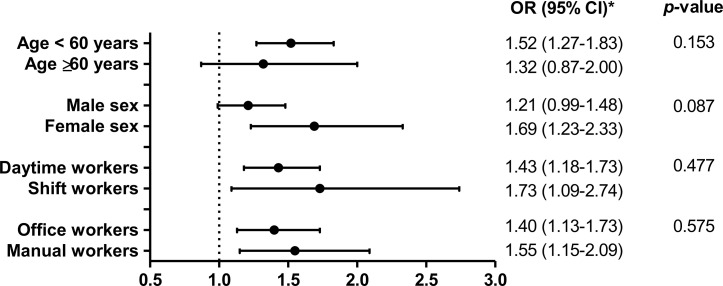
Association between working hours and NAFLD in subgroup analysis. *ORs for NAFLD in subjects working 53–83 hours/week in reference to those working the standard 36–42 hours/week are presented with 95% CIs.

Further logistic regression was performed with stratification of subgroups by age (< 60 *vs.* ≥60 years), sex (male *vs.* female), working schedule (daytime *vs.* shift workers), and occupation type (office *vs.* manual workers) (**Figure 2**). All p-values for interaction were not significant. No differences were observed according to working schedule or occupation type. The association between NAFLD and working hours was significant in subjects aged <60 years and in female subjects.

## Discussion

This nationally representative data showed that long working hours are associated with higher odds of NAFLD in workers without previous liver disease or heavy alcohol drinking habits. The prevalence of NAFLD increased as the working hours per week increased. Subjects who work 53–83 hours/week had 1.23 times higher OR for NAFLD than those working the standard 36–42 hours/week, independent of known risk factors for NAFLD.

Excessive working hours pose a serious danger to the health of workers. Concerns about the health status of workers are increasing, as many negative effects of long working hours are continuously being reported. In a meta-analysis, long working hours were associated with an increased risk of coronary heart disease and stroke compared with standard working hours (2). Besides the well-established risk for cardiovascular disease in individuals who work long hours, risks for various work-related metabolic diseases, such as obesity, hypertension, type 2 diabetes mellitus, and metabolic syndrome, have also been reported. Recently, Zhu et al. reported that long working hours are associated with obesity-related harmful outcomes in their meta-analysis (17). Although these metabolic diseases are closely related to NAFLD, no previous studies have addressed the association between long working hours and NAFLD. NAFLD is a clinical syndrome characterized by the accumulation of adipose tissue in the liver (18). It is rapidly becoming a major threat for liver-related deaths and a leading indication for liver transplantation (19), warranting the assessment of the risk factors for this disease entity. However, NAFLD is usually asymptomatic, making it difficult to identify every individuals having NAFLD in general population. Also, liver biopsy is regarded as a gold standard for the diagnosis of NAFLD, which is invasive and therefore may not be appropriate for large epidemiological studies. The HSI scoring system used in this study is a simple, noninvasive, and well-validated screening tool for NAFLD and thus, widely used in many previous studies to assess NAFLD (12–14).

One plausible factor that may explain the link between long working hours and NAFLD would be physical inactivity in overworking individuals. Working excess hours may limit leisure-time physical activity and may result in fat accumulation (20). Sedentary behavior is known to be associated with the risk of NAFLD development, and decreased physical activity is known to intensify the severity of NAFLD (21). Unhealthy dietary patterns may also play a role in increasing the risk for NAFLD. A previous study suggested that long working hours were associated with poor eating habits such as skipping breakfast, eating out, eating instant food, and overeating (22). These unhealthy diet patterns were found to be an independent risk factor for the onset of NAFLD (23). Long working hours are also associated with lifestyle habits such as smoking and alcohol drinking (24). However, the present study demonstrated a robust relationship between long working hours and NAFLD even after adjusting for confounding lifestyle factors. In addition, work-related sleep deprivation or chronic stress may be other underlying mechanisms contributing to the development of NAFLD by causing weight gain *via* alteration in hormones or energy homeostasis (25). A previous study reported that sleep duration was found to have a significant moderating effect on working hours and NAFLD (26). They observed that risk for NAFLD in long working hour group was higher than standard working hour group only in subjects sleeping 5 – 6 hours/day and not in those sleeping <5 or ≥7 hours/day. However, in our study, the negative effect of long working hours on NAFLD was significant even after adjusting for mean sleep duration of the subjects. Further studies are necessary to make a clear conclusion for this issue.

Different effects of long working hours on NAFLD were observed across subgroups. While, in subjects aged <60 years, long working hours amounting to 53–83 hours/week were associated with a significantly higher risk for NAFLD (OR 1.52, 95% CI 1.27–1.83) than working hours of 36–42 hours/week, the OR was statistically not significant (1.32, 95% CI 0.87–2.00) in a subgroup of subjects aged ≥60 years. According to an epidemiological review, NAFLD is more common in the younger to middle-aged groups, with a decline in prevalence at the age of 50–60 years (27). The small number of subjects aged ≥60 years who work 53–83 hours/week (7.1% of the study subjects) may have limited this subgroup to reach statistical significance. The OR for NAFLD was more pronounced in female workers (1.69, 95% CI 1.23–2.33) than in male workers (1.21, 95% CI 0.99–1.48). In Korean society, women tend to take more familial responsibilities as the primary caregiver for children and households (28). Thus, full-time working female employees may exhibit poorer health outcomes because of additional family work burden, including preparing meals, cleaning, and childcare (29). This may partially explain the higher risk in female workers. However, information on time spent for household jobs is not available in the current dataset and could not be analyzed. Although statistically not significant, shift workers had a higher OR for NAFLD than daytime workers. Shift work cycle-induced alterations of circadian rhythm have been shown to potentiate pro-inflammatory cytokine expression in adipose tissue as well as insulin resistance and glucose intolerance in high-fat diet-fed mice (30). Previous studies reported that shift work is associated with metabolic syndrome (31, 32). In this manner, shift workers may have higher risk for NAFLD. Office workers and manual workers had a relatively similar risk for NAFLD in the present study.

There are some limitations to address. First, because this was a cross-sectional study, the observed association does not necessarily reflect a causal relationship. Second, we used an indirect measure (HSI scoring system) rather than imaging or pathology examinations to define NAFLD. However, as previously mentioned, HSI is a well-validated scoring system for predicting the presence and degree of NAFLD in many large-scale studies. The prevalence of NAFLD according to HSI was 24.6% in this study cohort, which is comparable to the estimated prevalence of NAFLD worldwide, supporting the accuracy of HSI in determining NAFLD. Third, this study could not evaluate the association between long working hours and liver fibrosis by using NAFLD fibrosis score due to lack of data on certain parameters or fibrosis-4 score due to too small number of subjects having significant fibrosis based on the cut-off level of ≥2.67. Fourth, because this study included only the Korean population, the results of our study need to be confirmed in other ethnicities in the future. Lastly, although we attempted to adjust for various possible risk variables, there might be residual confounding factors or bias that we could not control, such as detailed dietary patterns including components of macro- and micronutrients.**** Despite these limitations, the present study had noteworthy strengths, including the nationally representative large sample size, standardized high-quality clinical and laboratory data collection, and comprehensive information about various confounding factors. Moreover, this study identified a significant association between long working hours and NAFLD, which may provide insight into new mechanisms and strategies for preventing NAFLD.

In conclusion, long working hours are associated with a higher risk for NAFLD than standard working hours. This association is more pronounced in workers aged <60 years and in female workers but is consistent regardless of working schedule or occupation type. Considering the wide range of adverse health outcomes of long working hours, protecting the health of workers through the regulation of working hours may be necessary, especially to reduce the risk of NAFLD. Further prospective studies are required to validate the causal relationship and to examine the effects of intervention of modulating the working hours.

## Data Availability Statement

Publicly available datasets were analyzed in this study. This data can be found here: http://knhanes.cdc.go.kr/knhanes/eng.

## Ethics Statement

The studies involving human participants were reviewed and approved by Korea University Institutional Review Board. The patients/participants provided their written informed consent to participate in this study.

## Author Contributions

ES, JK, JY, NanK, and KC conceptualized and designed this study. ES, ER, NamK, HY, JS, and SK acquired, analyzed, and interpreted the data. NanK, SB, and KC provided intellectual input into the interpretation of data. In addition, ES and KC drafted and revised this study. ES, JK, ER, and HY reviewed and edited the manuscript and contributed to the discussion. All authors contributed to the article and approved the submitted version.

## Funding

This work was supported by the Korea University Research Fund (K2100331).

## Conflict of Interest

The authors declare that the research was conducted in the absence of any commercial or financial relationships that could be construed as a potential conflict of interest.

The reviewer [HSC] declared a past co-authorship with one of the authors [KMC] to the handling editor.
