# Supplementary material for: Long Working Hours and Risk of Nonalcoholic Fatty Liver Disease: Korea National Health and Nutrition Examination Survey VII
Source: Front Endocrinol (Lausanne). 2021 May 6;12:647459. doi: 10.3389/fendo.2021.647459 (PMC8138556; doi:10.3389/fendo.2021.647459)

Supplementary Material

# Supplementary Table 1. Working patterns and sleep duration according to working hours.

|  | **36–42 hours/week (n = 2259)** | **43–52 hours/week**  **(n = 1885)** | **53–83 hours/week (n=1517)** | ***p*-value** |
| --- | --- | --- | --- | --- |
| **Working schedule** |  |  |  | **<0.001** |
| **Daytime** | **2010 (89.0%)** | **1658 (88.0%)** | **1246 (82.1%)** |  |
| **Afternoon** | **99 (4.4%)** | **64 (3.4%)** | **94 (6.2%)** |  |
| **Night** | **24 (1.1%)** | **30 (1.6%)** | **41 (2.7%)** |  |
| **Regular shifts** | **115 (5.1%)** | **119 (6.3%)** | **129 (8.5%)** |  |
| **Irregular shifts** | **11 (0.5%)** | **14 (0.7%)** | **7 (0.5%)** |  |
| **Type of employment** |  |  |  | **<0.001** |
| **Self-employed** | **310 (13.7%)** | **317 (16.8%)** | **525 (34.6%)** |  |
| **Employee** | **1856 (82.2%)** | **1511 (80.2%)** | **886 (58.4%)** |  |
| **Unpaid family worker** | **93 (4.1%)** | **57 (3.0%)** | **106 (7.0%)** |  |
| **Occupation** |  |  |  | **<0.001** |
| **Office worker** | **1656 (73.3%)** | **1326 (70.3%)** | **853 (56.2%)** |  |
| **Manual worker** | **603 (26.7%)** | **559 (29.7%)** | **664 (43.8%)** |  |
| **Sleep duration** |  |  |  | **<0.001** |
| **<5 hours/day** | **61 (2.7%)** | **53 (2.8%)** | **95 (6.3%)** |  |
| **5–6 hours/day** | **797 (35.3%)** | **717 (38.0%)** | **593 (39.1%)** |  |
| **≥7 hours/day** | **1401 (62.0%)** | **1115 (59.2%)** | **829 (54.6%)** |  |

# Supplementary Figures

## Supplementary Figure 1. Description of the study subjects


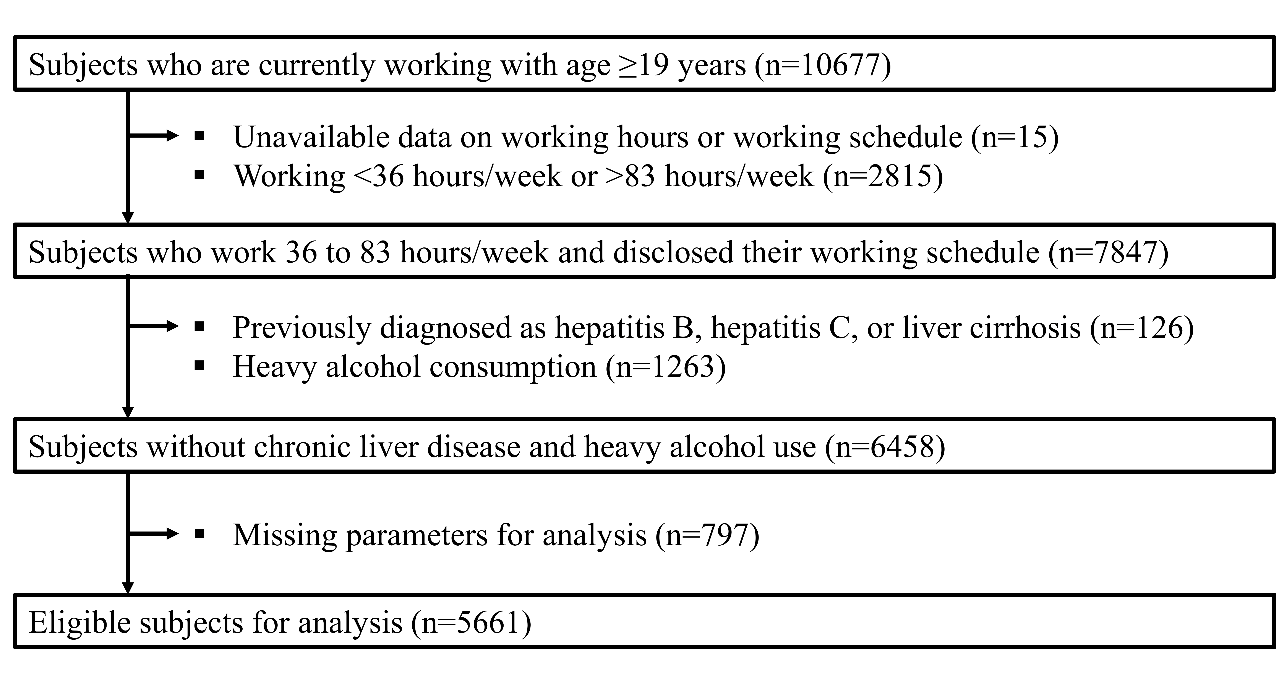


## Supplementary Figure 2. Association of working hours with (A) body mass index (BMI), (B) aspartate aminotransferase (AST), and (C) alanine aminotransferase (ALT) by quartiles.


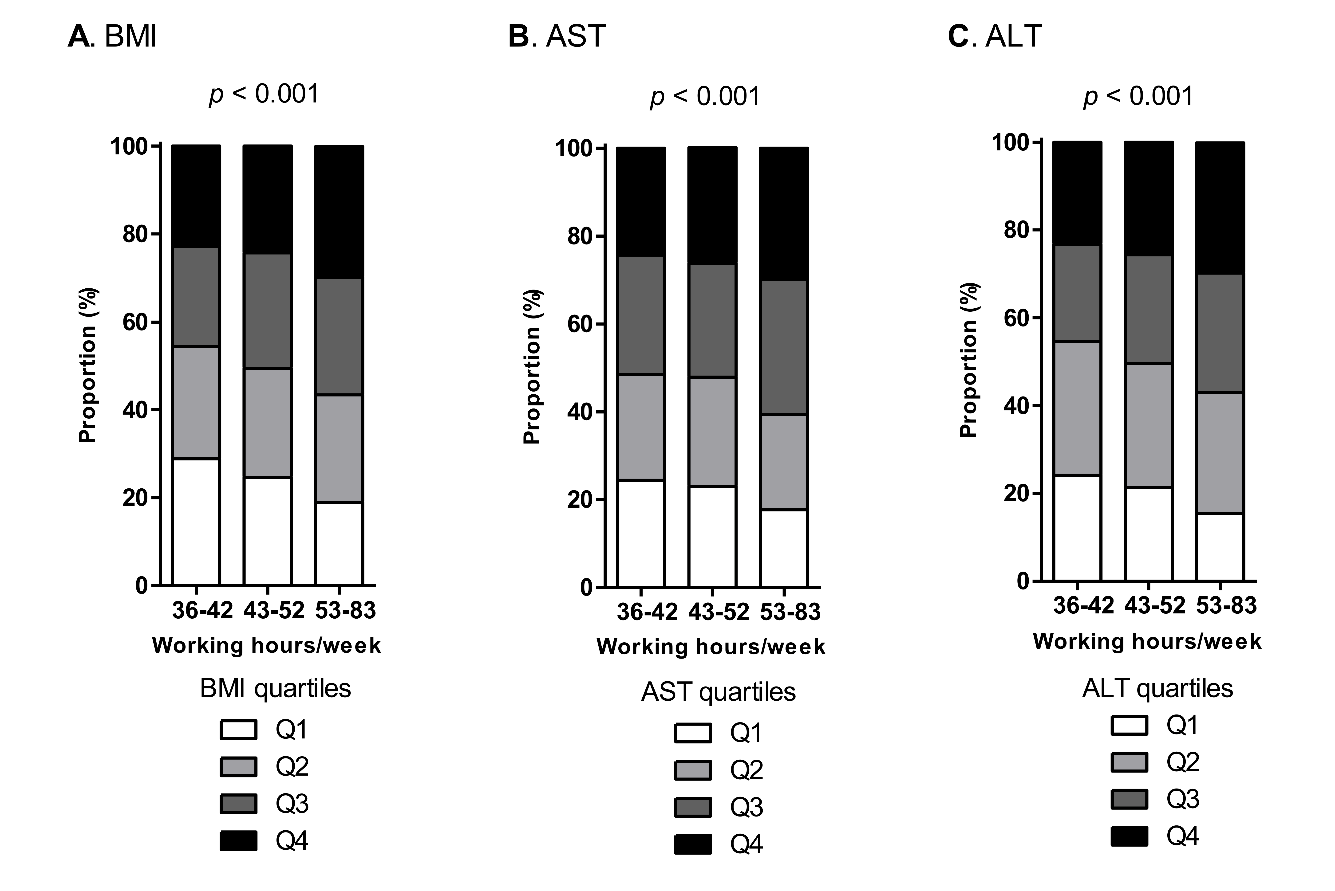

Supplement: Supplementary file 1 [file DataSheet_1.docx]
